# Starch Metabolism in Wheat: Gene Variation and Association Analysis Reveal Additive Effects on Kernel Weight

**DOI:** 10.3389/fpls.2020.562008

**Published:** 2020-10-06

**Authors:** Jian Hou, Yunchuan Liu, Chenyang Hao, Tian Li, Hongxia Liu, Xueyong Zhang

**Affiliations:** ^1^Key Laboratory of Crop Gene Resources and Germplasm Enhancement, Institute of Crop Sciences, Chinese Academy of Agricultural Sciences (CAAS), Beijing, China; ^2^State Key Laboratory of Plant Physiology and Biochemistry, College of Biological Sciences, China Agricultural University, Beijing, China

**Keywords:** wheat, starch metabolism, association study, kernel weight, starch content, amylose content, haplotype

## Abstract

Kernel weight is a key determinant of yield in wheat (*Triticum aestivum* L.). Starch consists of amylose and amylopectin and is the major constituent of mature grain. Therefore, starch metabolism in the endosperm during grain filling can influence kernel weight. In this study, we sequenced 87 genes involved in starch metabolism from 300 wheat accessions and detected 8,141 polymorphic sites. We also characterized yield-related traits across different years in these accessions. Although the starch contents fluctuated, thousand kernel weight (TKW) showed little variation. Polymorphisms in six genes were significantly associated with TKW. These genes were located on chromosomes 2A, 2B, 4A, and 7A; none were associated with starch content or amylose content. Variations of 15 genes on chromosomes 1A and 7A formed haplotype blocks in 26 accessions. Notably, accessions with higher TKWs had more of the favorable haplotypes. We thus conclude that these haplotypes contribute additive effects to TKW.

## Introduction

Wheat (*Triticum aestivum* L.) yield depends on three main components: spike number per area, grain number per spike, and thousand kernel weight (TKW). Because starch accounts for about 65–80% of the grain endosperm (Barron et al., 2007), starch metabolism can significantly affect TKW, yield, and quality (Keeling and Myers, 2010). Starch and sucrose metabolism involves several key enzymes: sucrose synthase (SUS), fructokinase (FRK), phosphoglucose isomerase (PGI), phosphoglucomutase (PGM), UDP-glucose pyrophosphorylase (UGPase), ADP-glucose pyrophosphorylase (AGPase), starch synthase (SS), granule-bound starch synthase (GBSS), starch branching enzyme (SBE), and debranching enzyme (DBE) (Jeon et al., 2010). Several transporters related to substrate and energy flow, such as ADP-glucose transporter (BT1), ATP/ADP transporter (AATP), glucose phosphate transporter (GPT), and triose-phosphate transporter (TPT), are rate-limiting for starch synthesis (Möhlmann et al., 1998; Bowsher et al., 2007). Enzymes involved in energy metabolism and starch degradation, including adenylate kinase (AK), beta-amylase (BMY), glucan, water dikinase (GWD), and phosphoglucan, water dikinase (PWD), also play important roles in grain filling (Stitt and Zeeman, 2012).

Starch consists of amylose starch and amylopectin starch (Hannah and James, 2008; Jeon et al., 2010), and functional changes in starch metabolism genes dramatically influence starch content, amylose content, and other agronomic traits. For instance, SUS is a key enzyme in the starch and sucrose synthesis pathway, and its overexpression can increase starch content in potato (*Solanum tuberosum* L.) and maize (*Zea mays* L.) (Baroja-Fernández et al., 2009; Li et al., 2013). ADP-glucose, a substrate of the SS isozymes, is mainly synthesized by AGPase. The activity of AGPase is correlated with starch content and grain weight (Smidansky et al., 2002; Kang et al., 2013; Tang et al., 2016). Amylose in the grain is mainly synthesized by GBSSI, and mutations in *GBSSI* can reduce amylose contents in rice (*Oryza sativa* L.), maize, barley (*Hordeum vulgare* L.), and wheat (Tsai, 1974; Sano, 1984; Nakamura et al., 1995; Patron et al., 2002; Perez et al., 2019). Notably, three wheat lines carrying null mutations in *GBSSI*, *SSIIa*, and *SBEIIa* produce starch with amylose contents of 0%, 46%, and 79%, respectively, compared with 34% in wild type (WT) (Botticella et al., 2018). In addition, mutations in *BT1* can affect starch content and the structure of starch granules (Li et al., 2017; Wang et al., 2019). Overexpression of plastidic AATP in potato can increase the accumulation of ADP-glucose and increase starch content by 16% to 36% compared with WT (Tjaden et al., 1998; Geigenberger et al., 2001). Decreasing the expression of *GWD* via RNAi can increase grain number per plant and TKW in wheat (Ral et al., 2012). Therefore, altering starch metabolism genes represents a promising approach by which to alter wheat grain components.

Although these genes have been widely studied, systematic studies on their functional variation are lacking, especially for wheat accessions. Here, we sequenced 87 genes involved in starch metabolism, using high-throughput sequencing. We identified over 8,000 variants in 300 wheat accessions and tested them for association with thousand kernel weight (TKW), starch content (SC), and amylose content (AC), to identify useful polymorphisms among the natural populations. Notably, variants of 15 genes located on chromosomes 1A and 7A formed haplotype blocks in one subgroup. Multiple variations of genes were associated with TKW, starch weight per grain (SW), and amylose weight per grain (AW). The favorable haplotypes had positive additive effects on TKW. These results provide insights relevant to the analysis of gene function, and the variations we detected might be valuable in wheat production.

## Materials and Methods

### Plant Materials

Three hundred wheat accessions were used in the study: 115 Chinese landraces (CLs) and 55 modern Chinese cultivars (MCCs) from the mini-core collection (Hao et al., 2011), 127 foreign cultivars (FCs), and three tetraploid wheat cultivars (Supplementary Table S1). All accessions were provided by the Institute of Crop Sciences, CAAS. These accessions were grown in the order of MCCs, CLs and FCs in the same block at Xinxiang Experiment Station in Henan Province, China, in 2017 and 2018. Each accession was planted in four rows, at a planting density of 40 seeds per row. The length of each row was 2 m, and the rows were 25 cm apart. The two native cultivars Zhengmai 9023 (ZGNKE087A) and Yanzhan 1 (ZGNKE088A) were used as the check cultivars and were planted after every 50 accessions (200 rows). The thousand kernel weight (TKW) was measured for eight plants from the middle row of each of the CL and MCC accessions. The TKWs of FCs and tetraploid wheat were not measured in this study, because these accessions flowered too late.

### Starch and Amylose Content Assay

Mature grains (100 g) of CL and MCC accessions were processed into flour with a Quadrumat Senior mill (Brabender, Germany). The flour for each accession was then processed to quantify starch content by the AOAC Method 996.11 (McCleary et al., 1997), using the Total Starch (AA/AMG) Assay Kit (K-TSTA-50A, Megazyme, Ireland), and the amylose content was assessed using the Amylose/Amylopectin Assay Kit (K-AMYL, Megazyme, Ireland). Starch weight per grain was calculated as (TKW/1000) × flour yield × starch content. Amylose weight per grain was calculated as starch weight per grain × amylose content.

### Genotype Detection

All accessions were genotyped by the MolBreeding Biotechnology Co., Ltd. (Shijiazhuang, China). The genome-specific primers were designed by the GenoPlexs method (MolBreeding). The PCR reaction contained 50 ng DNA, 10 μL GenoPlexs 2 × G Master Mix and 8 μL primer mixture. PCR was carried out using an ABI 9700 machine with the following settings: denaturation at 95°C for 5 min, followed by 19 cycles of 95°C for 30 s, annealing at 60°C for 8 min, and extension at 72°C for 5 min. The DNA concentrations were measured by Qubit Fluorometric Quantitation (Thermo Fisher, Shanghai), and the DNA library was sequenced by BGI (Shenzhen, China). FastQC was used for quality control of the data^1^. Sequences were aligned with GATK^2^ to detect polymorphic sites relative to the reference genome of Chinese Spring (IWGSCv1.0)^3^.

### Genetic Structure, Association Analysis, and Statistical Analysis

The genetic structure of the cultivar set was evaluated by Structure 2.3.4 (Pritchard et al., 2000) using 8,141 SNP markers sequenced in this study. The number of subgroups was determined by the ΔK model (Evanno et al., 2005). Principal coordinate analysis (PCA) was performed to reveal relationships among different groups using TASSEL 5 (Bradbury et al., 2007), and three groups were identified by plotting the first two axes. Association analysis was carried out using the MLM (mixed linear model) module (Q + K) of TASSEL 5. A K matrix was generated by the Kinship module of TASSEL 5. SNP loci with minor allele frequencies lower than 0.05 or a missing ratio higher than 0.1 were not considered, and the threshold *P* of association signals was set as the 0.05/SNP marker number (0.05/1,102 = 4.537 × 10^–5^); namely, -log_10_ *P* > 4.343. The agronomic traits and genetic effects of favorable haplotypes in different groups were tested via ANOVA in SPSS 21.0. Pearson’s correlation coefficient was calculated between pairs of variables using the data per accession. Correlations were tested for significance using the *F*-test at a significance level of 1% (*P* < 0.01).

### Evolutionary Studies

Diversity analysis of different genomes was carried out using DnaSP version 6 (Rozas et al., 2017). *F*_ST_ tests for the 87 genes were carried out with Arlequin 3.5.1.2 (Excoffier and Lischer, 2010). The sequences of the genes from Chinese Spring (CS) were used as a comparison.

## Results

### Polymorphisms Within Genes Involved in Starch Metabolism

For each of the 300 accessions, 87 genes (Supplementary Table S1) involved in starch metabolism were amplified using 285 pairs of genome-specific primers (Supplementary Table S2) and were then sequenced by high-throughput sequencing (Supplementary Table S3). The amplified sequences aligned to 1,090,267 bp in the CS reference genome^4^. For each gene, the amplified region contained the entire coding region, from 2.5 kb upstream of the start codon to 500 bp downstream of the stop codon. In total, 8,141 polymorphic sites were detected from the amplified regions among all accessions (Supplementary Table S4): 2,858 in the A genome, 2,843 in the B genome, and 2,440 in the D genome. The nucleotide diversities (π) of genes from the A, B, and D genomes were 0.002918, 0.002439, and 0.001149, respectively (Supplementary Figure S1A). The diversity of the D genome was the lowest: the mean minor allele frequency (MAF) of the D genome was only 0.027 (Supplementary Table S4), which suggested that most variations in the D genome were rare alleles. These polymorphic sites were mainly located in the introns and 5’ UTRs (Supplementary Figure S1B). The A genome contained the highest density of polymorphic sites (7.4 per kb) within coding regions compared with the other two genomes (6.5 and 6.2 per kb; Supplementary Figure S1C). Within the coding regions of the 87 genes, approximately half of the variations were synonymous mutations (SS; Supplementary Figure S1D); the others included 402 non-synonymous mutations (NS) and 73 insertion/deletions (InDels). These NSs and InDels could be useful for functional analyses of these 87 starch metabolism-related genes.

### Genetic Structure of Accessions

**FIGURE 1:**
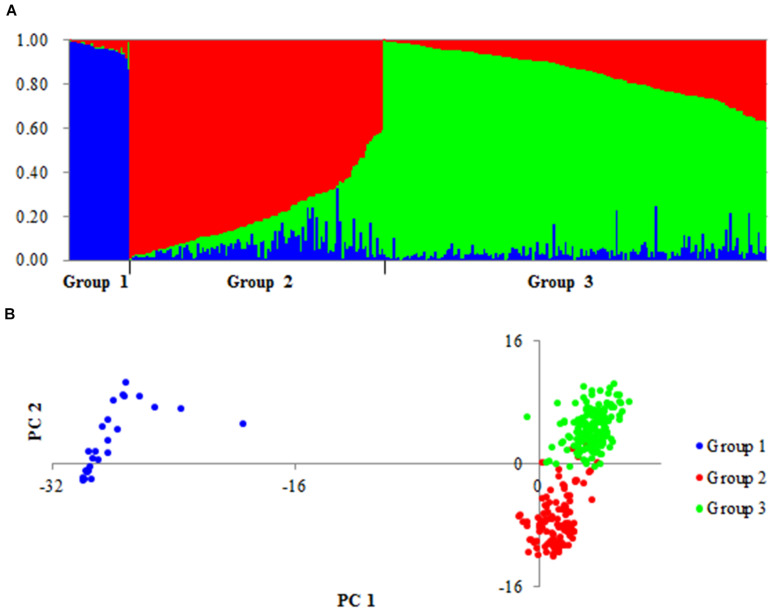
Population structure of 300 accessions based on 8,141 variations. **(A)** Genetic structure of three groups produced by structures 2.3.4. **(B)** PCA plots of all accessions based on the same number of markers.

On the basis of the polymorphic sites identified above, the accessions were separated into Groups 1, 2 and 3 (Figure 1). Accessions of Group 1 consisted of 20 Chinese landraces (CLs) and six foreign cultivars (FCs). Accessions of Group 2 consisted of 81 CLs, 18 modern Chinese cultivars (MCCs), and 10 FCs. Accessions of Group 3 consisted of 14 CLs, 37 MCCs, and 114 FCs. Therefore, Groups 1 and 2 were mainly landraces, and Group 3 contained mainly modern cultivars. Notably, principal coordinate analysis (PCA) suggested that Groups 1 and 2 possessed very different genetic backgrounds (Figure 1).

**FIGURE 2:**
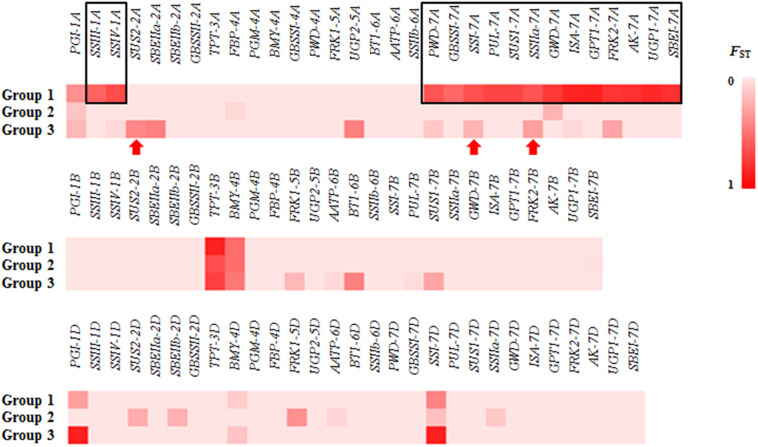
The *F*_ST_ for all of the genes in different groups. Large differences in *F*_ST_ between Group 1 and Group 2/3 were detected at 15 genes (black rectangle). Red arrow: gene associated with thousand kernel weight (TKW) had a different *F*_ST_ in Group 3.

To validate the large difference between Groups 1 and 2, the genetic differentiation (*F*_ST_) of different groups was detected for all genes in the three groups. Large differences in *F*_ST_ between Groups 1 and 2/3 were detected in 15 genes: 2 genes on chromosome 1A (*SSIII-1A* and *SSIV-1A*) and 13 genes on 7A (Figure 2). In Group 1, the SNPs of 15 genes formed haplotype blocks that were genetically distinct from other random haplotypes in Groups 2/3 (Supplementary Figure S2). Given this huge genetic difference, accessions of Group 1 might be useful for the genetic modification of modern cultivars.

### Comparison and Correlation of Six Traits in Three Groups

**FIGURE 3:**
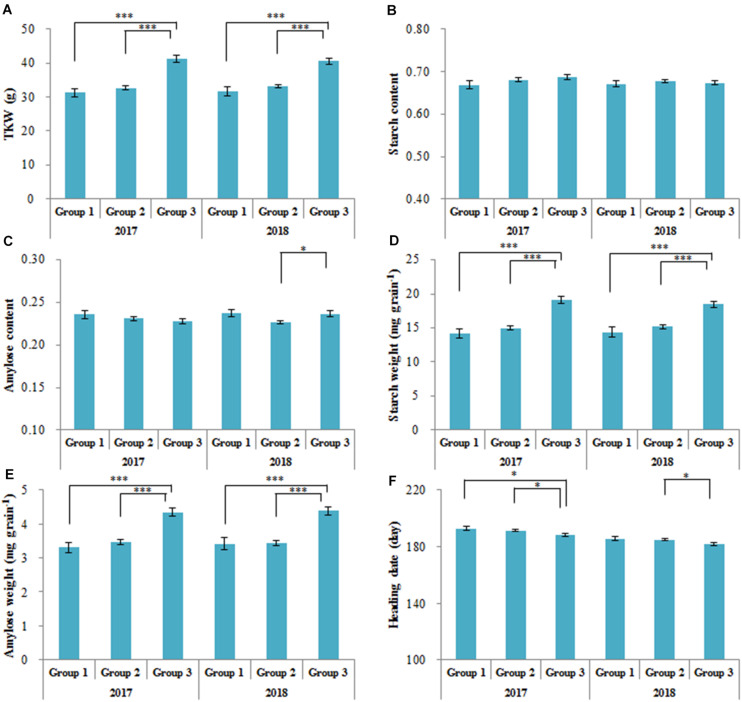
Agronomic traits of three groups (as in Figure 1) in 2017 and 2018. **(A)** Thousand kernel weight (TKW); **(B)** starch content; **(C)** amylose content; **(D)** starch weight per grain; **(E)** amylose weight per grain; **(F)** heading date. ^∗^*P* < 0.05; ^∗∗∗^*P* < 0.001.

Thousand kernel weight (TKW), starch content (SC) and amylose content (AC), starch weight per grain (SW), amylose weight per grain (AW), and heading date (HD) of Chinese accessions from the three groups were measured in 2017 and 2018 (Figure 3). The traits of FC and tetraploid wheat were not measured in this study because these accessions flowered too late. The mean TKW, SW, and AW of Group 3 were significantly higher than those of Groups 1/2 in 2017 and 2018 (*P* < 0.001), but SC did not differ significantly among the three groups. A significant difference in AC was detected only between Groups 2 and 3 in 2018 (*P* < 0.05). Significant differences in HD existed between Groups 1/2 and 3 (*P* < 0.05), but the greatest difference in HD between Groups 1 and 3 was only 4.4 days, in 2017.

**FIGURE 4:**
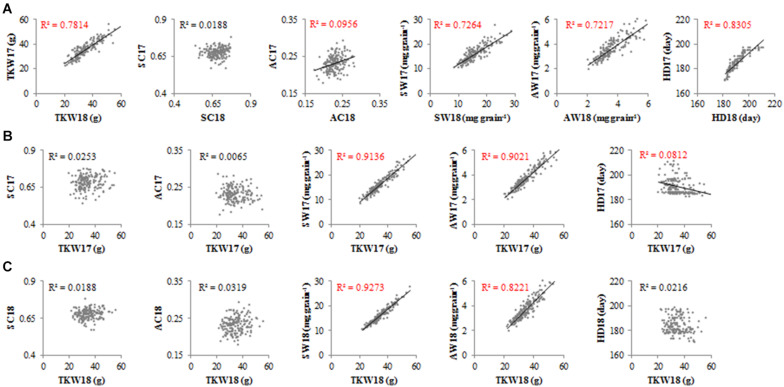
Correlation between traits for all accessions in 2017 and 2018. TKW, thousand kernel weight; SC, starch content; AC, amylose content; SW, starch weight per grain; AW, amylose weight per grain; HD, heading date. **(A)** Correlation between the same traits in 2017 and 2018. **(B)** Correlation between TKW and other traits in 2017. **(C)** Correlation between TKW and other traits in 2018.

The TKW of accessions fluctuated slightly across different years (Figure 4A). The SC-2017 (SC17) did not correlate with SC-2018 (SC18), although AC-2017 (AC17) correlated with AC-2018 (AC18) in the accessions (*R*^2^ = 0.0956). The SW, AW, and HD exhibited the same trends as TKW in 2017 and 2018. The TKW was correlated with SW and AW, but TKW was correlated not with SC, AC (Figures 4B,C), or HD18. These results suggest that TKW, SW, AW, and HD fluctuated little across different years, but SC varied in an irregular manner.

### Association Analysis With TKW, SW, and AW

**FIGURE 5:**
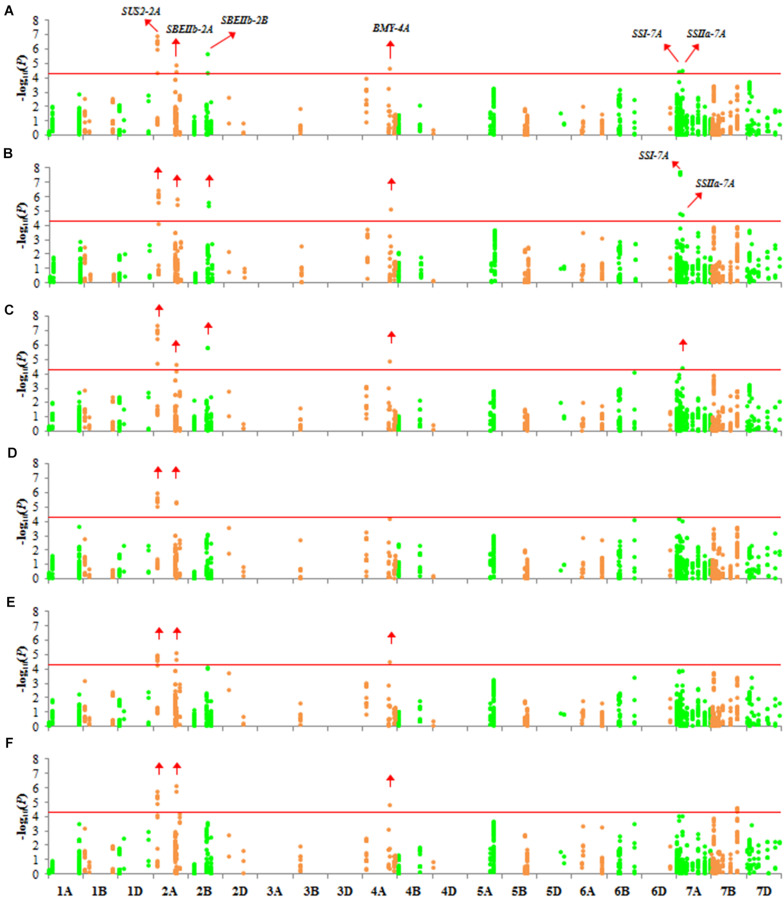
Manhattan plots for TKW, SW, and AW with 1,102 markers with MLM (Q + K) at *P* < 4.537 × 10^–5^. The red horizontal line corresponds to the threshold value for significant association. Green and orange colors represent different chromosomes. Significantly associated genes are labeled with red arrows. **(A)** TKW in 2017; **(B)** TKW in 2018; **(C)** SW in 2017; **(D)** SW in 2018; **(E)** AW in 2017; **(F)** AW in 2018.

To clarify whether the detected genetic variations influenced TKW, 1,102 variations were selected to test for their association with TKW (MAF > 0.05 and missing ratio < 10%). The significance threshold was given as *P* < 0.05/1102; namely, -log_10_ *P* > 4.343. Six genes (*SUS2-2A*, *SBEIIb-2A*, *SBEIIb-2B*, *BMY-4A*, *SSI-7A*, and *SSIIa-7A*) were associated with TKW in 2017 and 2018 (Figures 5A,B). Association signals were mainly distributed on the A genome (chromosomes 2A, 4A, and 7A). Because SW and AW had high correlation coefficients with TKW, the genes associated with SW and AW were similar to those associated with TKW (Figures 5C–F).

Because significant differences in TKW were observed between Group 1/2 and Group 3, we examined differences in *F*_ST_ of Group 3. Three genes associated with TKW had a different *F*_ST_ in Group 3 (Figure 2, red arrows). Among these genes, we detected three non-synonymous mutations, located in the second exon of *SUS2-2A* (GGG → GAG, chr2A: 121145639), and in the second (AAC → AAG, chr7A: 77440026) and eleventh (AAC → AGC, chr7A: 77435977) exons of *SSI-7A*.

### Association Analysis With SC and AC

**FIGURE 6:**
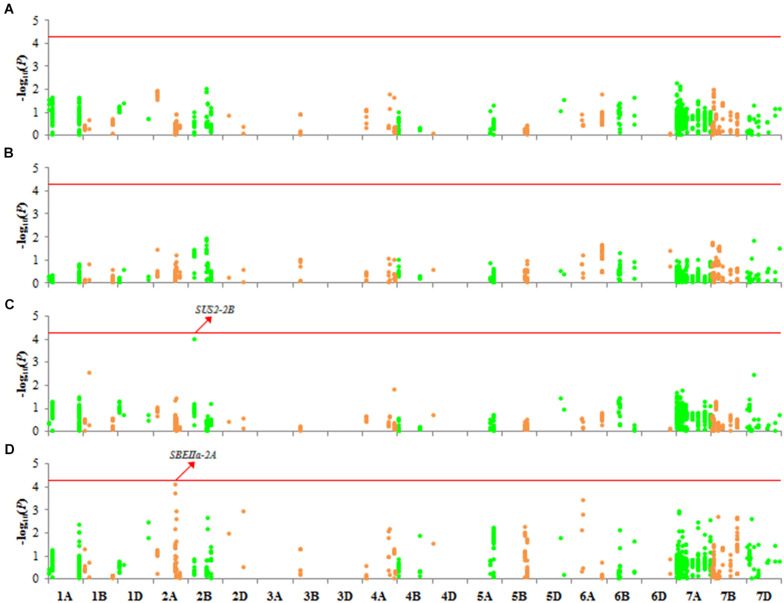
Manhattan plots for SC and AC with 1,102 markers with MLM (Q + K) at *P* < 4.537 × 10^–5^. **(A)** SC in 2017; **(B)** SC in 2018; **(C)** AC in 2017; **(D)** AC in 2018.

We detected no variation associated with SC or AC (Figure 6). However, association signals (-log_10_ *P*) of loci on two genes, *SUS2-2B* and *SBEIIa-2A*, were above 4 with AC (Figures 6C,D). *SBEIIa-2A* plays a role in the amyloplast. However, these association signals were either in introns or in the UTRs, and therefore, these genes influence AC requires further verification.

### Six Genes Have an Additive Effect on TKW

**FIGURE 7:**
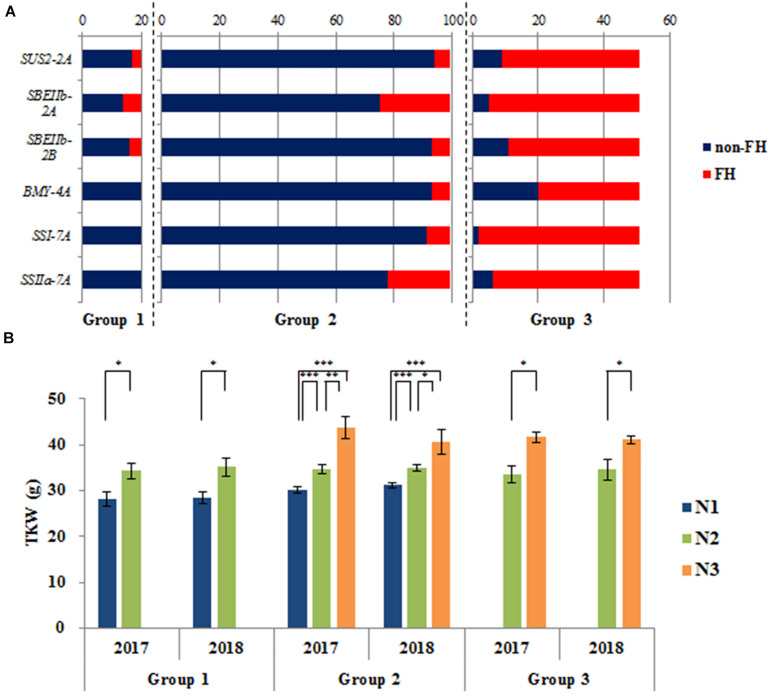
The additive effects of six genes on TKW. **(A)** Components of favorable haplotypes (FH) of six genes in three groups in Chinese accessions; **(B)** TKW of different numbers of favorable haplotypes (NFHs) in three groups in Chinese accessions. N1 (NFH 0), N2 (NFH 1–3), and N3 (NFH 4–6). *P < 0.05; **P < 0.01; ***P < 0.001.

We detected six genes associated with TKW. Variations in these genes mainly comprised two haplotypes: CS types and non-CS types. For the six genes, non-CS types were favorable haplotypes associated with a higher TKW. Among the three groups, the haplotype frequencies of these genes differed markedly (Supplementary Table S3). The materials were classified into three groups based on the number of favorable haplotypes (NFH), namely N1 (NFH 0), N2 (NFH 1–3), and N3 (NFH 4–6). The mean NFH values in the three groups were 1.1, 0.9, and 4.8, respectively. In Chinese accessions, the frequencies of favorable haplotypes varied widely in Group 3 compared with those in Group 1/2 (Figure 7A), which suggested that favorable haplotypes underwent selection in the breeding process. Accessions of N3 had the highest TKW among all accessions (Figure 7B). Notably, the greater the NFH in an accession, the greater the TKW; this pattern suggests that the favorable haplotypes of these genes had additive effects on TKW.

## Discussion

Previous research on single genes involved in starch metabolism has often failed to explain substantial changes in phenotype (Weichert et al., 2010; Cakir et al., 2016). In this study, we sequenced 87 genes involved in starch metabolism from 300 wheat accessions and measured six traits (TKW, SC, AC, SW, AW, and HD) of CL and MCC in 2017 and 2018. We detected no significant correlation between starch content (SC) over different years (Figure 4). These results are similar to the findings of Fahy et al. (2018), who reported no significant correlation between the SC of mature or developing grains in 2013 and 2014. We conclude that SC varies irregularly and may be easily influenced by environmental factors.

Studies of SC or amylose content (AC) in cereal crops have mainly relied on functional mutational analysis and QTL mapping. In rice, an A-to-G change that results in an Asp^165^/Gly^165^ substitution at GBSSI led to a low AC in a Yunnan rice landrace (Liu et al., 2009). Several mutations in *TaAGP.L-B1* and *TaSSIVb-D* genes resulted in different expression patterns of *TaGBSSII* (upregulated), *TaAGPSS*, *TaSSI*, and *TaSBEII* (downregulated) and led to a reduction in starch and amylopectin contents (Zhang S. L. et al., 2019). In a wheat double haploid (DH) population derived from Huapei 3 and Yumai 57, the two QTLs *QTsc4A.1* and *QAms4A.1* were associated with SC and AC at five stages under three treatments in two seasons, and they also were expressed from 12 to 22 DAF (Tian et al., 2015). In the natural population examined here, we identified only two genes (*SUS2-2B* and *SBEIIa-2A*) that may be associated with AC (Figure 6); their function in affecting the amylose content requires further study. In addition, 15 genes located on chromosomes 1A and 7A had very different variations in Group 1 (Figure 2 and Supplementary Figure S2). PCA similarly revealed that accessions of Group 1 had different genetic backgrounds (Figure 1), which suggested they might origin from different tetraploid ancestors. Several of these 15 genes, including *SSIII-1A*, *SSIV-1A*, *GBSSI-7A*, *SSI-7A*, *SSIIa-7A*, *ISA-7A* and *SBEI-7A*, encoded enzymes that are functional in the amyloplast. Group 1 was not associated with SC or AC, but this might due to the small size of Group 1 (only 20 Chinese landraces were measured in the study). A DH or recombinant inbred (RIL) population derived from accessions in Groups 1 and 2/3 could be used to analyze these specific variations further.

In this study, six genes associated with TKW were mainly associated with SW and AW, which suggests that optimizing starch metabolism might improve TKW in wheat. Different haplotypes containing key mutations could lead to trait changes (Deng et al., 2018). Three non-synonymous mutations in *SUS2-2A* and *SSI-7A*, which form different haplotypes, could be developed into functional markers for high TKW. However, functional mutations in natural populations are limited. Several transcription factors affect starch biosynthesis in rice and maize (Peng et al., 2014; Huang et al., 2016; Zhang et al., 2016; Wu et al., 2019; Zhang Z. Y. et al., 2019; Deng et al., 2020). However, only a few transcription factors have been reported to affect starch biosynthesis in wheat and include, for example, *TabZIP28* and *TaRSR1* (Liu et al., 2016; Song et al., 2020). Novel transcription factor mining might represent a useful direction by which to improve starch synthesis in wheat.

We previously studied the artificial selection of genes that influence TKW, such as *TaSUS1* and *TaSUS2* (Hou et al., 2014), *TaAGPL* and *TaAGPS1* (Hou et al., 2017), *TaCWI* (Jiang et al., 2015), *TaBT1* (Wang et al., 2019), *TaGW2* (Qin et al., 2014), *TaGS5* (Ma et al., 2016), and *qKW-6A* (Chen et al., 2019). In this study, six important genes associated with TKW were analyzed to uncover further the frequency changes of haplotypes and their additive effects on TKW (Figure 7). The favorable haplotypes were probably selected from landraces to produce modern cultivars. Because the effects of favorable haplotypes are mainly additive, these variations could help guide the design of functional markers to assist in breeding for high yield.

## Conclusion

We sequenced 87 genes involved in starch metabolism in 300 wheat accessions and identified SNPs associated with TKW, SC, AC, SW, and AW. The TKW, SW, and AW fluctuated little across different years, but SC varied irregularly. Six genes were associated with TKW, SW, and AW, which could be exploited for high TKW. The favorable haplotypes had positive additive effects on TKW.

## Data Availability Statement

The datasets presented in this study can be found in online repositories. The names of the repository/repositories and accession number(s) can be found in the article/ Supplementary Material.

## Author Contributions

JH performed the experiments and data analysis and prepared the manuscript. YL contributed to collecting and measuring phenotypic data in 2017 and 2018. CH, TL, and HL provided experimental material. XZ, the corresponding author, conceived the original research and revised the manuscript. All authors contributed to the article and approved the submitted version.

## Conflict of Interest

The authors declare that the research was conducted in the absence of any commercial or financial relationships that could be construed as a potential conflict of interest.

## Footnotes

1 www.bioinformatics.babraham.ac.uk/projects/fastqc

2 software.broadinstitute.org/gatk

3 http://www.wheatgenome.org/

4 https://wheat-urgi.versailles.inra.fr/Seq-Repository

## Supplementary Material

Supplementary Figure 1 Polymorphic sites of all the 87 genes. **(A)** Nucleotide diversity (π) of the A, B, and D genome; **(B)** numbers of polymorphic sites at different regions of all genes; **(C)** density of polymorphic sites at the coding regions on the A, B, and D genome; **(D)** numbers of three types of mutations: SS, synonymous mutations; NS, non-synonymous mutations; InDels, insertions/deletions.

Supplementary Figure 2 Haplotypes of 15 genes located on chromosomes 1A and 7A. Horizontal lines represent variations within each gene (light blue, CS type; orange, non-CS type); vertical lines represent accessions in each group. Chromosomes 1A and 7A (left, short arm; right, long arm) are at the top. 1, *SSIII-1A*; 2, *SSIV-1A*; 3, *PWD-7A*; 4, *GBSSI-7A*; 5, *SSI-7A*; 6, *PUL-7A*; 7, *SUS1-7A*; 8, *SSIIa-7A*; 9, *GWD-7A*; 10, *ISA-7A*; 11, *GPT1-7A*; 12, *FRK2-7A*; 13, *AK-7A*; 14, *UGP1-7A*; 15, *SBEI-7A*.

Supplementary Table 1 Genes used in this study and their chromosome locations.

Supplementary Table 2 Primers used in this study. Primers were designed according to the CS v1 reference sequence.

Supplementary Table 3 Accessions and their agronomic traits in 2017 and 2018.

Supplementary Table 4 Polymorphic sites detected in all accessions.
