# Supplementary material for: Starch Metabolism in Wheat: Gene Variation and Association Analysis Reveal Additive Effects on Kernel Weight
Source: Front Plant Sci. 2020 Oct 6;11:562008. doi: 10.3389/fpls.2020.562008 (PMC7573188; doi:10.3389/fpls.2020.562008)
Supplement: Supplementary Figure 1 — Polymorphic sites of all the 87 genes. (A) Nucleotide diversity (π) of the A, B, and D genome; (B) numbers of polymorphic sites at different regions of all genes; (C) density of polymorphic sites at the coding regions on the A, B, and D genome; (D) numbers of three types of mutations: SS, synonymous mutations; NS, non-synonymous mutations; InDels, insertions/deletions. [file Data_Sheet_1.PDF]

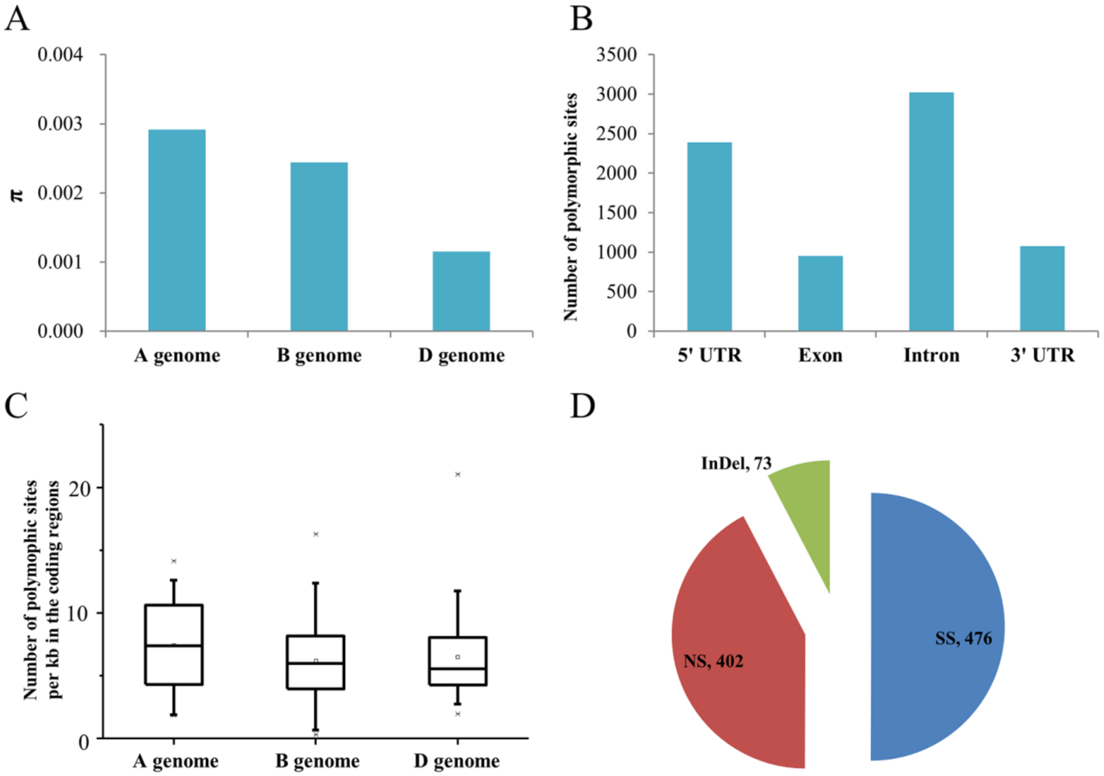

Figure S1. Polymorphic sites of all the 87 genes. (A) Nucleotide diversity (π) of the A, B and D genome; (B) numbers of polymorphic sites at different regions of all genes; (C) density of polymorphic sites at the coding regions on the A, B and D genome; (D) numbers of three types of mutations: SS, synonymous mutations; NS, nonsynonymous mutations; InDels, insertions/deletions.

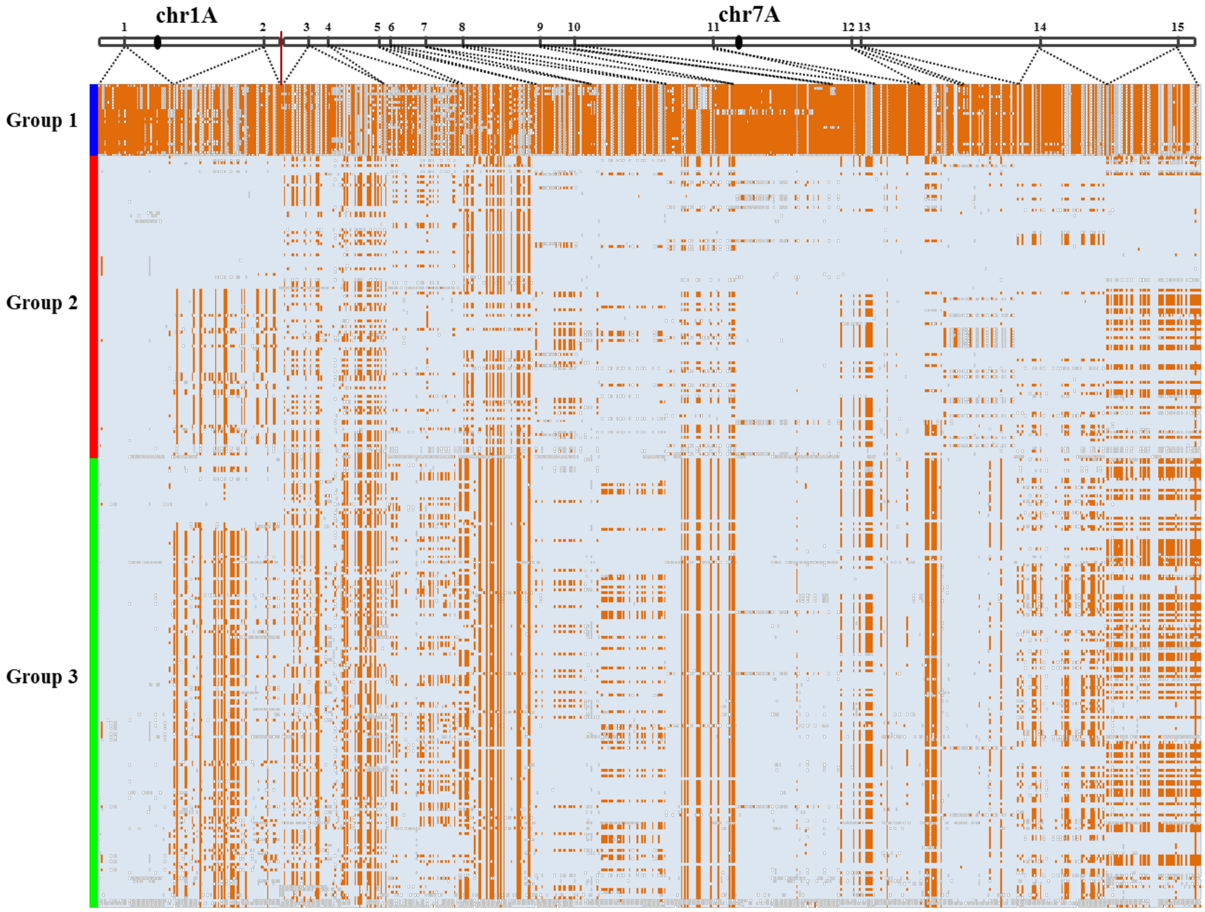

Figure S2. Haplotypes of 15 genes located on chromosomes 1A and 7A. Horizontal lines represent variations within each gene (light blue, CS type; orange, non-CS type); vertical lines represent accessions in each group. Chromosomes 1A and 7A (left, short arm; right, long arm) are at the top. 1, *SSIII-1A*; 2, *SSIV-1A*; 3, *PWD-7A*; 4, *GBSSI-7A*; 5, *SSI-7A*; 6, *PUL-7A*; 7, *SUS1-7A*; 8, *SSIIa-7A*; 9, *GWD-7A*; 10, *ISA-7A*; 11, *GPT1-7A*; 12, *FRK2-7A*; 13, *AK-7A*; 14, *UGP1-7A*; 15, *SBEI-7A*.
